# 

**Published:** 1847-12

**Authors:** 


### CONTENTS

OF NO. VI.

### ORIGINAL COMMUNICATIONS.

ESSAYS AND CASES.

ART. I.—Notes on Medical Matters and Medical Men in Paris. By DAVID W. YANDELL, M.D., of Louisville, Ky.........463

ART. II.—Clinical Cases. Reported by BENJ. F. WENDEL, M.D....479

ART. III.—A Case of Four Children at a Birth, complicated with Hemorrhage. By JOHN H. DONNELL, Student of Medicine in the University of Louisville..........................483

ART. IV.—Removal of a large Scrotal Tumor. By LEWIS A. SAYRE, M.D., of New York..................................485

ART. V.—Cases. By Dr. T. C. OSBORNE, of Erie, Ala...........487

ART. VI.—Pseudo-Membranous Cynanche Trachealis, following Scarlatina Simplex. By Dr. M. H. FEE, Hiltonsville, Ia.....493

BIBLIOGRAPHICAL NOTICES.

ART. VII.—Lectures on Natural and Difficult Parturition. By EDWARD WILLIAM MURPHY, A.M., M.D., Professor of Midwifery, University College, London; Obstetric Physician, University College Hospital; and formerly Assistant Physician to the Dublin Lying-in Hospital. New York: S. S. and William Wood......................................497

SELECTIONS FROM AMERICAN AND FOREIGN JOURNALS.

Puerperal Fever................................................511

Nature of Intestinal Secretions in Cholera.........................515

Sketch of Burmah and Medical Science among the Burmese........515

Diamond Converted into Coke ....................................529

Medical Heroism........530
Report on the Influence of Light on the Growth of Plants........534
Lectures of M. Agassiz in New York........535
Case of Delirium Tremens treated by Inhalation of Ether, at the Seamen's Retreat, Staten Island........537
On some recent and remarkable Examples of the Protection afforded by Metallic Conductors against heavy Strokes of Lightning........539
On the Time required to produce Death by a fatal dose of Medicinal Hydrocyanic Acid........540
Sulphur Rains........544
Ledoyen's Disinfecting Fluid........545

ORIGINAL INTELLIGENCE.

To Subscribers, Readers, and Contributors........547
Proposed Treatise on Typhoid and Typhus Fever........548
Epidemic Cholera........549
Alleged Extirpation of the Liver........549
University of Louisville........550
Books Received........550
Odd Numbers Wanted........550

